# Host Epigenetic Alterations and Hepatitis B Virus-Associated Hepatocellular Carcinoma

**DOI:** 10.3390/jcm10081715

**Published:** 2021-04-16

**Authors:** Mirjam B. Zeisel, Francesca Guerrieri, Massimo Levrero

**Affiliations:** 1 Cancer Research Center of Lyon (CRCL), UMR Inserm 1052 CNRS 5286 Mixte CLB, Université de Lyon 1 (UCBL1), 69003 Lyon, France; francesca.guerrieri@inserm.fr; 2 Hospices Civils de Lyon, Hôpital Croix Rousse, Service d’Hépato-Gastroentérologie, 69004 Lyon, France

**Keywords:** hepatitis B virus, hepatocellular carcinoma, HBx, virus–host interactions, epigenetic regulation, epidrugs

## Abstract

Hepatocellular carcinoma (HCC) is the most frequent primary malignancy of the liver and a leading cause of cancer-related deaths worldwide. Although much progress has been made in HCC drug development in recent years, treatment options remain limited. The major cause of HCC is chronic hepatitis B virus (HBV) infection. Despite the existence of a vaccine, more than 250 million individuals are chronically infected by HBV. Current antiviral therapies can repress viral replication but to date there is no cure for chronic hepatitis B. Of note, inhibition of viral replication reduces but does not eliminate the risk of HCC development. HBV contributes to liver carcinogenesis by direct and indirect effects. This review summarizes the current knowledge of HBV-induced host epigenetic alterations and their association with HCC, with an emphasis on the interactions between HBV proteins and the host cell epigenetic machinery leading to modulation of gene expression.

## 1. Introduction

Hepatocellular carcinoma (HCC), the most frequent primary malignancy of the liver, is among the leading causes of cancer-related deaths worldwide [1]. The World Health Organization (WHO) estimates that one million individuals will die from liver cancer per year until 2030. The vast majority of HCCs are associated with chronic liver disease due to a known underlying etiology, including chronic viral hepatitis (B and C), alcohol intake, and metabolic diseases [2]. Importantly, treatment of the underlying cause, e.g., inhibition of hepatitis B virus (HBV) replication or eradication of hepatitis C virus (HCV), reduces but does not eliminate the risk of HCC development.

Common mechanisms and pathways involved in HCC development have been described for these etiological factors [3,4]. Liver cirrhosis is an important risk factor for HCC but some HCCs can develop in the absence of cirrhosis, in particular for HCCs associated with metabolic steatohepatitis and HBV-related HCCs [2]. HCC is an extremely heterogenous cancer both at the histological and molecular levels (reviewed in [3,4]). Several subclasses/subtypes have been described based on histological features, transcriptomic data and genetic alterations [3,5]. Heterogeneity is not only observed between tumors of different patients but also between cancer cells within the same tumor nodule [4,6].

The clinical outcome of HCC is dismal with a 5-year survival rate of 18% [7]. HCC is one of the most chemo-resistant tumor types and treatment options are limited. Curative treatments include percutaneous ablation, surgical resection or liver transplantation but are limited to patients with early disease. Unfortunately, tumors are often diagnosed at a late stage and thus the majority of HCC patients are not eligible for these procedures but can benefit from systemic approaches. The multikinase inhibitor (MKI) sorafenib has long been the only option for HCC treatment in patients with advanced disease. Much progress has been made in HCC drug development in the past few years. Several other MKIs (lenvatinib, regorafenib, cabozantinib), the anti-vascular endothelial growth factor (anti-VEGF) receptor(R)2 antibody ramucirumab and immune checkpoint inhibitors (ICIs) are being tested or have become alternative first-line (lenvatinib) or second-line therapies (regorafenib, cabozantinib, ramucirumab, nivolumab, pembrolizumab) [8]. The success of combination therapy targeting both VEGF (bevacizumab) and PD-L1 (atezolizumab) as front-line treatment in the IMbrave150 study [9] has, very recently, led to its adoption as the first-choice treatment in first-line therapy for advanced HCC [10]. Other combinations of ICIs and ICIs plus MKIs are being tested, raising the expectations for increased survival in HCC patients with advanced disease [10,11].

The major cause of HCC is chronic HBV infection, which the WHO estimates to affect more than 250 million individuals globally. While HBV infection can be prevented by a vaccine, there is to date no cure for chronic hepatitis B. Indeed, a viral cure is hampered by the persistence of the viral genome in the nucleus of infected cells as a stable episome (called covalently closed circular DNA (cccDNA)) that is not targeted by standard-of-care antiviral therapy. HBV contributes to liver carcinogenesis by direct and indirect effects. In addition to triggering chronic liver inflammation and immune response-mediated liver necrosis, HBV plays a direct role in HCC development by inducing genomic instability/insertional mutagenesis following integration of viral DNA into the host genome at defined sites, including TERT and CCNE1 genes [12,13], and by affecting various cellular functions via viral proteins (reviewed in [3]).

Both the HBV replication cycle and viral pathogenesis are tightly linked to epigenetic processes. The HBV cccDNA is associated with cellular histones to form a viral minichromosome. The viral proteins HBc and HBx are incorporated into the viral minichromosome and it is now well established that transcription from cccDNA depends on both HBx and the cellular transcriptional machinery with post-translational modifications of histones within the cccDNA chromatin playing a key role in the transcriptional regulation of the viral episome [14,15,16,17,18,19]. Furthermore, a comprehensive genome and methylome analysis of HCC tissues indicated that initial HBV integration appears to preferentially occur at active chromatin areas, while selection processes during clonal evolution may lead to a more frequent detection of clonal integration sites in methylated/closed chromatin areas [20]. Finally, viral proteins have been shown to be recruited to the host chromatin and to influence gene transcription by modulating epigenetic modifications and interacting with transcription factors, chromatin-modifying enzymes and the basal transcriptional machinery [3,21]. A better understanding of these mechanisms will ultimately contribute to design novel therapeutic strategies towards an HBV cure and for HBV-related HCC.

In this review, we summarize the current knowledge of HBV-induced host epigenetic alterations and their association with HCC.

## 2. Epigenetic Mechanisms in HCC

**Figure 1:**
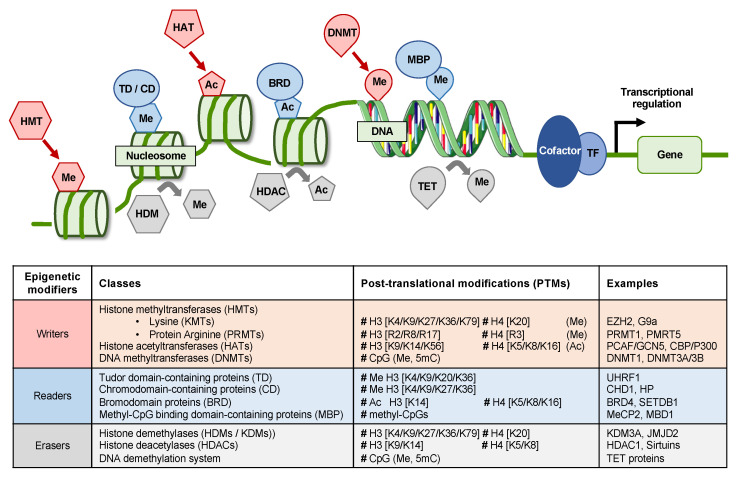
Schematic representation of epigenetic writers (i.e., histone methyl-transferases (HMTs), histone acetyl-transferases (HAT), DNA methyltransferases (DNMT)), readers (e.g., TD, CD, BRD proteins, MBP) and erasers (i.e., HDM, HDAC, TET). Ac: acetylation; CD: chromodomain-containing protein; DNMT: DNA methyltransferase; HAT: histone acetyltransferase; HDAC: histone deacetylase; HDM: histone demethylase; TF: transcription factor; MBP: methyl CpG binding protein; Me: methylation; TD: Tudor domain-containing proteins; TET: Ten-eleven translocation. Images were adapted from SMART (Servier Medical Art).

Epigenetics refers to the study of dynamic heritable changes in gene expression that are not due to modification of the DNA sequence. Epigenetic mechanisms regulate chromatin conformation and recruitment of the transcriptional machinery as well as regulatory molecules, thereby modulating gene expression. Epigenetic effectors include chromatin remodeling complexes, DNA methylation/demethylation enzymes, histone modification enzymes, histone mark readers and noncoding RNAs. Epigenetic modifiers can be classified into epigenetic writers that add a covalent modification to DNA or histones (e.g., DNA methyltransferases (DNMTs), histone acetyl-transferases (HATs) and histone methyl-transferases (HMTs)), erasers that remove these epigenetic marks (e.g., histone demethylases (HDMs) or deacetylases (HDACs)) as well as readers that recognize specific marks (e.g., bromodomain (BRD)-containing proteins) and enable subsequent modification of gene expression by recruiting additional factors (Figure 1). Given the reversibility of epigenetic modifications, epigenetic modifiers represent interesting targets for drug design.

**Figure 2:**
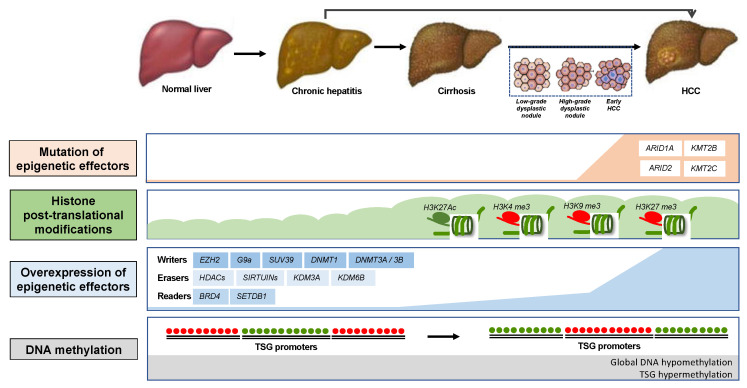
Schematic representation of alterations of epigenetic modifiers, chromatin modifications and DNA methylation during hepatocarcinogenesis. TSG: tumor suppressor gene.

By affecting major cell functions, alterations of DNA methylation, chromatin modification and ncRNAs can contribute to various aspects of hepatocarcinogenesis. Mutations of epigenetic modifiers are frequent in HCC [22,23]. A recent study analyzing data from 365 patients with HCC derived from The Cancer Genome Atlas (TCGA) reported that mutations and changes in expression of epigenetic modifiers are common events in HCC that lead to an aggressive gene expression program and poor clinical prognosis [24]. Indeed, more than 75% of patients exhibit a mutation of at least one epigenetic modifier and 20% of them have mutations of more than five epigenetic modifiers [24]. Alterations appear to be more frequent in lysine MTs (KMTs) and lysine MDs (KDMs) than in HATs or BRD proteins [24]. The increasing knowledge about the epigenetic mechanisms involved in liver carcinogenesis (for recent reviews see [25,26,27,28]) (Figure 2) opens perspectives for novel therapeutic approaches for HCC treatment.

## 3. Deregulation of Epigenetic Mechanisms by HBV

**Figure 3:**
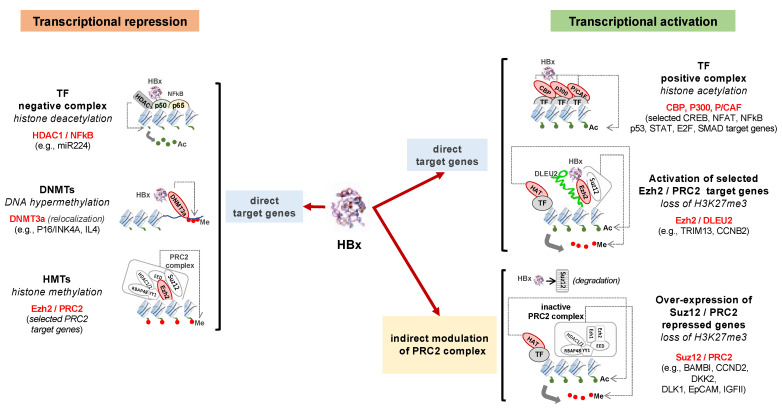
Schematic illustration of host gene transcription modulation by HBx. Examples of HBx interactions with the host cell epigenetic machinery and their effects on host gene transcription are shown. A description of HBx/PRC2 target genes are detailed in [33]. Ac: acetylation; DNMT: DNA methyltransferases; HAT: histone acetyltransferase; HMT: histone methyltransferase; HBx: hepatitis B virus (HBV) protein x; HDAC: histone deacetylase; TF: transcription factor; Me: methylation.

In addition to their roles in the HBV replication cycle and cccDNA minichromosome assembly and/or activity, the viral proteins HBc and HBx have been shown to interact with several host proteins, RNA as well as DNA and to deregulate cellular functions (reviewed in [3,16,29]), including modulation of host gene expression [21,30,31,32,33]. By interfering with major cellular processes, these viral proteins may contribute to both viral escape from host immune responses and cellular transformation. HBx has been shown to bind to promoters of a wide variety of genes involved in cell metabolism, chromatin dynamics and cancer as identified by ChIP followed by sequencing (ChIP-Seq) analysis in HBV-replicating human hepatoma HepG2 cells [21]. Interestingly, in addition to protein-coding genes, HBx was also found in promoter regions of lncRNA and miRNA, among which ncRNAs were involved in the regulation of liver functions and were hijacked by the virus to boost its replication and carcinogenesis [21]. HBc has also been reported to directly bind to selected host gene regulatory regions, including genes of innate immune responses [30,31,32,34,35,36]. By combining chromatin immunoprecipitation (ChIP) and location analysis with genome-wide tiling arrays (ChIP-on-ChIP), Guo et al. generated a human genome-wide binding profile of HBc using hepatocytes isolated from HBV patient liver biopsies [37]. HBV core protein was found at the promoter regions of more than 3000 genes, among which were genes involved in metabolic processes and regulatory pathways [37]. It has to be noted, however, that subsequent attempts to generate a ChIP-Seq genome-wide repertoire of host chromatin regions bound by HBc have failed so far (Testoni B and Levrero M, unpublished results). Further studies are needed to understand how HBc regulates host cell gene expression and the consequences for HBV pathogenesis. Some of the molecular mechanisms underlying HBV protein-mediated interactions with the host cell epigenetic machinery and modulation of gene expression are detailed below and summarized in Figure 3.

### 3.1. Alteration of Host DNA Methylation

DNA methylation is mostly found on the fifth position of cytosine (5mC) in CpG dinucleotides and is frequently associated with closed chromatin conformation/inhibition of transcription. Aberrant DNA methylation—consisting of DNA hypomethylation and/or promoter gene CpG hypermethylation—is associated with many different cancers, including HCC. While global DNA hypomethylation is associated in cancer and in HCC with the activation of protooncogenes and increased genome instability, hypermethylation on CpG islands located in the promoter regions of tumor suppressor genes results in their transcriptional silencing and increases tumor risk. Several epigenetically silenced putative tumor suppressor genes have been found in HCC tissues as well as nontumor tissues from HCC patients, suggesting that aberrant methylation occurs both at early and late stages of malignant transformation of the liver [38]. Interestingly, the methylation pattern of defined subsets of genes has been associated with HCC poor prognosis including in HBV-associated HCC patients [38,39]. Furthermore, it has been shown that HBV-associated HCC displays a distinct methylation pattern different to HCC due to other etiologies [40,41,42].

DNA tumor viruses are known to manipulate host DNA methylation to alter expression of immune-related genes, which may create an environment favorable for viral escape from the host immune system as well as for cancer cell evasion from antitumor immune responses (reviewed in [43]). Not surprisingly, HBV infection is associated with aberrant methylation [44,45,46,47,48,49]. DNA hypermethylation is mediated by DNA methyltransferases (DNMTs) that possess de novo methylation activity. In line with its pleiotropic activities, HBx has been shown to promote global hypomethylation as well as specific regional hypermethylation of tumor suppressor genes by deregulating DNMTs [44]. Indeed, expression of HBx in liver-derived cell lines increases DNMT activity, in line with increased DNMT1 and DNMT3A levels [44,50,51]. Conversely, HBx has been reported to decrease DNMT3B expression [44]. Furthermore, HBx-transgenic mice display, before the full development of HCC, a severe hypomethylation of intragenic CpG islands (mCGIs), a subset of CpG islands that are normally highly methylated by the DNMT3L complex and marked with epigenetic signatures associated with active expression, such as H3K36me3 [45]. Hypomethylation of mCGIs is caused by Dnmt3L and Dnmt3a downregulation following the direct binding of HBx and HDAC1 to their promoters and leads to the downregulation of many developmental regulators that could facilitate tumorigenesis [45]. Of note, by analyzing HCC patient-derived liver tissues, Park et al. observed that genomic hypomethylation was more frequent and pronounced in HBx-positive HCC versus HBx-negative HCC [44], further supporting the notion that HBx likely contributes to aberrant methylation patterns in vivo. Interestingly, HBx can directly interact with DNMT3A and the recruitment of HBx–DNMT3A complexes to regulatory promoters of defined genes led to their silencing while the transcription of other genes was activated due to the redistribution of DNMT3A [52]. HBx has been also shown to enhance DNMT1 and DNMT3A recruitment to the promoter to silence the expression of the ankyrin-repeat-containing, SH3-domain-containing, and proline-rich-region-containing protein family 2 (ASPP2)—a mediator of p53 and p53 family member apoptosis—that is downregulated in HCC cell lines and in the livers of HBV-related HCC patients [53]. HBx-directed recruitment of DNMT3A and DNMT3B to CpG island 1 within the promoter of metastasis-associated protein 1 (MTA1) has been found to inhibit the binding of p53 to the MTA1 promoter and its p53-mediated repression, resulting in an increased expression of MTA1, enhanced invasiveness and metastasis of HCC [54]. Similarly, the recruitment of DNMT1 and DNMT3a to the promoters of the secreted frizzled-related protein SFRP1 and SFRP5 is facilitated by HBx, leading to their downregulation in hepatoma cells and HCC patients [55]. Tumor suppressor genes regulated by HBx-modulated DNA methylation events include SOCS-1, RASSF1A, procadherin-10 (PCDH10), insulin-like growth factor-binding protein 3 (IGFBP3) and E-cadherin [56,57,58,59,60].

In contrast to DNA methylation catalyzed by DNMTs, demethylation of DNA can either occur through dilution of 5mC during replication in the absence of methylation maintenance or be catalyzed in a sequential process involving different players. The ten-eleven translocation (TET) family of dioxygenases oxidize 5mC to 5-hydroxymethylcytosine (5hmC), which can be further oxidized to 5-formylcytosine (5fC) and 5-carboxylcytosine (5caC). 5fC and 5caC can then be either diluted during replication or be removed by thymine DNA glycosylase (TDG) and be replaced by cytosine. Of note, oxidized forms of 5mC appear to be more than DNA demethylation intermediates and have been shown to possess regulatory functions (reviewed in [61,62]). A recent study reported that the global 5hmC and 5fC contents decreased in HCC tissues as compared to peritumor tissues and that HBV infection exacerbated these differential levels of 5hmC and 5fC in patient-derived liver tissues [49]. The effect of HBV on modified cytosine DNA contents was also shown using cell-based models and HBx seems to play a role in this process [49]. Notably, decreased 5hmC and 5fC genomic DNA contents in HCC tissues were associated with poor prognosis [49].

### 3.2. Modification of Histone Marks

Acetyl moiety cycling on lysine residues of histones is catalyzed by HATs and HDACs. HATs can be divided into four families based on their primary structures: GNAT (Gcn5, PCAF, Hat1, Elp3 and Hpa2), p300/CBP (p300 and CBP), MYST (Esa1, MOF, Sas2, Sas3, MORF, Tip60 and Hbo1) and Rtt10. HDACs comprise zinc-dependent HDACs (HDAC1-11) and non-zinc-dependent HDACs or sirtuins (Sirt1-7) and their activities have been associated with silencing of gene expression. Overexpression of different HDACs has been reported in HCC tissues but with conflicting data between patient cohorts [63,64]. HBx has been shown to interact with HDAC1 using IP assays [52,65]. While Zheng et al. reported that HBx did not alter HDAC1 expression, HDAC catalytic activity, total histone acetylation patterns, and acetylated H3 or H4 distribution patterns in defined gene promoters, including IGFBP3, CDH6, MT1F and IL4R in Huh7-based models [52], other studies using Hep3B- and HepG2-based models indicated that HBx recruits HDAC1 to the promoters of CDH1 and IGFBP3 and that there are decreased levels of acetyl-H3 and RNA polymerase II at the CDH1 promoter [60,65]. HBx also binds SIRT1 and disrupts its interaction with *β*-catenin that is then free to transactivate cancer-promoting genes such as cyclin-D1 and c-myc [66]. HBx has also been shown to interact with p300/CBP to increase p300 recruitment to the IL8 and PCNA promoters and thereby increase gene expression in liver-derived cells [67]. Moreover, the HBx-p300/CBP interaction induces MBD2-HBx-CBP/p300 complex formation, which contributes to the hypomethylation and transcriptional activation of the IGF-II P3 and P4 promoters and CBP/p300-mediated acetylation of histones H3 and H4 in HepG2 and Huh7 cells [68]. This is in line with studies showing increased IGFII expression in HCC tissues from HBV-related HCC patients [69].

Similar to HATs and HDACs, HMTs and HDMs are responsible for the cycling of methyl groups on lysine (KMTs) and arginine (RMTs) residues in histone tails. Canonical lysine methylation sites on core histones comprise H3K4, H3K9, H3K27, H3K36, H3K79, and H4K20, each of them having a different role in determining the functional features of chromatin. Methylations of H3K9 and H3K27 are generally described as repressive markers, whereas H3K4, H3K36, and H3K79 usually designate regions of active chromatin [70] and H4K20 mono-, di-, or tri-methylation have distinct genomic distributions and functions, with an opposite impact on transcription. Several KMTs, including EZH2, SETDB1, EHMT2/G9a and SUV39h1, are frequently upregulated in HCC and high expression levels of these enzymes have been associated with poor prognosis [71,72,73,74,75,76]. Interestingly, several studies have reported an interplay between KMTs and HBV in different model systems. Wang et al. performed ChIP combined with genome tiling arrays (ChIP-on-chip) to profile H3K9me3 enrichments on gene promoters in HepG2 hepatoma cells transfected with HBx and found alteration of H3K9me3 enrichments on promoters of genes that may be involved in tumorigenesis and cancer progression [77]. Notably, H3K9me3 and HBx staining in HCC tissues was significantly higher than those in adjacent nontumor tissues from HBV-related HCC patients [77]. HBx has also been shown to interact with the PSET and SET domains of SUV39h1 and to increase its HMT activity in HepG2 cells [78]. SUV39h1 expression was increased in human hepatocytes following infection of human liver-chimeric mice with HBV and in HCC tissues from HBV-related HCC patients [78]. HBx was shown to cooperate with SUV39h1 to increase the expressions of ATF6, AFP, GADD45a, and DUSP1, known to be involved in carcinogenesis and HCC development [78]. Since SUV39h1-mediated H3K9 tri-methylation has been consistently linked to gene silencing, the observed transcriptional activation of cancer-related genes is likely an indirect consequence of the induction of SUV39h1 activity by HBx rather than the result of its recruitment to those target genes. An indirect activation of transcription by SUV39h1 has been reported in melanoma tumorigenesis where H3K9 tri-methylation facilitates the DNMT3A-dependent methylation of the retinoblastoma (RB)1 promoter, leading to the release of E2F1 to activate the expression of the PIN1 peptidyl-prolyl cis-trans isomerase and RAF1-MEK-ERK signaling [79]. Altogether, the mechanistic basis for HBx-SUV39H1 interplay remains to be elucidated. HBx has been also reported to interfere with Polycomb Repressive Complex 2 (PRC2) involved in H3K27me3-mediated gene expression silencing [33,80,81]. The interplay between HBx and PRC2 is mediated by the activation and/or binding to host lncRNAs and will be discussed in detail in the following section. Interestingly, HBx has recently been shown to promote genome-wide H3K4 tri-methylation by stabilizing WDR5 [82], a core subunit of the human MLL and SET1 (hCOMPASS) H3K4 methyltransferase complexes that facilitates the assembly of hCOMPASS and other chromatin-modifying complexes [83]. HBx also has been shown to upregulate SET and MYND domain-containing 3 (SMYD3) H3K4 methyltransferase expression, leading to transactivation of oncogenes, such as c-myc in HepG2 cells [84].

Arginine methylation of histone (and nonhistone) proteins by protein arginine methyltransferases (PRMTs) plays a key role in epigenetic regulation of transcription, being implicated in both transcriptional activation and repression, as well as in other fundamental cellular processes including pre-mRNA splicing, DNA damage signaling, mRNA translation, cell signaling and cell fate decision [85]. PRMT family members tend to be upregulated in both hematological and solid tumors, contributing through multiple mechanisms to tumorigenesis, invasion and metastasis [85,86]. In HCCs, PRMT1, PRMT2, PRMT5 and PRMT9 overexpression is frequent and associated with advanced disease and/or poor prognosis [87,88,89,90,91,92,93,94], whereas PRMT6 downregulation was linked to metabolic reprogramming, sorafenib resistance and stemness [95,96]. Altered PRMT expression was not unequivocally associated with a specific risk factor, with the exception of PMRT9 overexpression found more frequently in HBV-related HCCs [94]. Although PMRT5-mediated symmetric di-methylation of cccDNA H4R3 results in the suppression of viral transcription and replication [97] and HBx has been shown to relieve the inhibitory effect of cccDNA-bound PMRT1 on the HBV minichromosome [98], whether and how HBV directly affects PMRT functions on the host chromatin have not been investigated so far.

### 3.3. Modulation of ncRNAs

ncRNAs are RNA transcripts that do not translate into proteins but contribute to regulate diverse cellular processes, including gene expression, through interactions with DNA, RNA or proteins. They are commonly subdivided into short ncRNAs (<200 nucleotides), which include miRNAs and long ncRNAs (>200 nucleotides) [99]. HCC metastasis, invasion, dissemination, and recurrence have been associated with altered expression of ncRNAs [100,101,102,103,104], suggesting that ncRNAs play an important role in liver carcinogenesis (for review see [105,106,107]). While miRNAs usually interact with mRNAs to regulate their stability/translation, lncRNAs, due to their structural complexity, can interact with DNA, RNA, and/or proteins and regulate gene expression by different mechanisms, including epigenetic silencing, splicing regulation, miRNA sponging, lncRNA–protein interaction, and genetic variation (reviewed in [107]). Not surprisingly, given their pleiotropic role in gene expression regulation, ncRNAs contribute to the regulation of the expression and/or function of the epigenetic modifiers described above. Whereas some ncRNAs appear to be frequently altered in many cancers including HCC, other ncRNAs can be differentially expressed depending on the HCC etiology [107] or the stage of liver disease and may thus represent diagnostic tools and/or therapeutic targets [108]. Furthermore, ncRNAs have been associated with HCC treatment efficacy/drug resistance [109].

As shown for other viruses, HBV infection is associated with modulation of host miRNA and lncRNA expression and numerous miRNAs have been reported to be deregulated in HBV-related HCCs in liver (tumor) tissues and/or serum/plasma (reviewed in [108]). HBV can directly regulate defined ncRNA levels and viral infection can also indirectly affect ncRNA levels. Some of these ncRNAs may affect the viral replication cycle (either positively or negatively) and/or contribute to liver pathogenesis (reviewed in [106,110]). miR-122, one of the most abundant miRNAs in the liver and hepatic tumor suppressor, directly binds a conserved region of the HBV pregenomic RNA, subsequently leading to inhibition of HBV replication [111]. In turn, HBV infection was shown to downregulate miR-122 expression, thereby promoting liver carcinogenesis [111]. Indeed, down-regulation of miR-122 occurs mainly in HBV-related HCC but not in HCV-related HCC in line with the negative and positive effects of miR-122 on the HBV and HCV life cycles, respectively (reviewed in [106]). Interestingly, the genome-wide binding profile of HBx in HBV-transfected HepG2 cells showed that this viral protein can be found in promoter regions of both miRNAs and lncRNAs [21], indicating that HBx directly modulates host ncRNA transcription. For example, HBx has been shown to upregulate the oncomiR miR-21 and promote HCC progression by targeting the tumor suppressor PTEN and programmed cell death protein-4 (PDCD4) [21,112,113]. Upregulation of miR-21 by HBx is mediated by both its binding to miR-21 promoter [21] and via IL6 induction and STAT3 activation [114].

Furthermore, HBx directly upregulated the lncRNA DLEU2 that has been shown to coregulate with HBx several host genes that may play a role in HCC [33]. Indeed, the HBx–DLEU2 interaction at host promoters has been shown to relieve EZH2-mediated gene repression and to lead to the transcriptional activation of a subset of EZH2/PRC2 target genes in HBV-infected cells and HBV-related HCCs [33]. Interestingly, other lncRNAs induced by HBx can modulate gene repression via PRC2—e.g., UCA1, which represses p27 expression by recruiting EZH2 [115]. Furthermore, HBx has been reported to reduce the expression of SUZ12, a PRC2 subunit, via PLK1 in collaboration with another HCC-related lncRNA, HOTAIR, suggesting a complex interplay between HBx, lncRNA and histone modifications [80].

## 4. Potential of Targeting Epigenetic Alterations in HBV-Associated HCC

The compounds aiming at the pharmacological restoration of epigenetic regulations by targeting epigenetic writers, readers and erasers in order to interfere with DNA methylation or post-translational histone modifications, are referred to as epidrugs or epigenetic drugs. The first epidrugs received FDA approval for the treatment of hematological malignancies more than 10 years ago and several new compounds are under clinical investigation in various cancers (reviewed in [28,116,117,118]), including HCC (Table 1). Preclinical and clinical evidence support the use of epidrugs in combination with chemotherapy, TKIs and ICIs (reviewed in [116,119]).

The anti-HCC effect has been mechanistically linked in preclinical models to decreased stemness and/or the induction of a differentiated phenotype but also to the restoration of sensitivity to sorafenib and/or to the potentiation of the effect of sorafenib or ICIs [28]. Thus, the pan-HDAC inhibitor resminostat was shown to induce the reversion of stem-like properties in HCC cells [120] and the HDAC inhibitor belinostat was reported to improve the antitumor activity of anti-cytotoxic T lymphocyte antigen 4 (CTLA4) antibody in combination or not with anti-programmed cell death protein 1 (PD-1) therapy in a subcutaneous Hepa129 murine HCC model [121].

Few studies have tested epidrugs in HCC patients (Table 1). The first-generation DNMT inhibitor 5-aza-2′-deoxycytidine (decitabine) has shown, at low doses, a favorable toxicity profile and some activity in patients with advanced HCC [122]. Results of clinical efficacy in HCC therapy have also been reported for the HDAC inhibitors belinostat and reminostat in combination with sorafenib [123,124]. Interestingly, resminostat has been shown to restore sorafenib sensitivity in the phase 1/2 SHELTER study [124]. Finally, the second-generation DNMT inhibitor guadecitabine has been evaluated in a phase 2 trial for patients with advanced HCC who failed prior sorafenib treatment (NCT01752933) and its association with the ICI durvalumab is currently being tested (NCT03257761).

Of note, HBV infection is often among the exclusion criteria in clinical trials evaluating epidrugs in HCC patients and there is no clinical trial specifically designed to test epidrugs in HBV-induced HCC. Conversely, several studies have reported data on epidrugs in HBV-induced HCC using cell culture-based models and mouse models (Table 2). In addition to the desired effect of defined epidrugs on host gene expression, some epidrugs can also act on HBV transcription. While some may decrease viral parameters [125], others have been shown to promote HBV replication [126,127,128,129] and may thus not be interesting drug candidates for HBV-related HCC.

Interestingly, recently a four-gene signature was reported to identify HCC patients from the TCGA database and HBV-associated HCC patients with poor prognosis that might potentially benefit from treatment with inhibitors of Jumonji-C-domain-containing (JmjC) histone demethylase [24].

In addition to epidrugs, ncRNAs also represent interesting therapeutic targets for chronic liver diseases (reviewed in [106,107,136]) as demonstrated by first proof-of-concept studies assessing the effect of miR-122 antagonists in chronic HCV-infected patients [137,138]. A miR-34 mimic (MRX34, Mirna Therapeutics) has been evaluated in patients with primary liver cancer in a phase I trial but the study was terminated due to serious immune-related adverse events (clinical trial NCT01829971).

## 5. Conclusions

HBV contributes to HCC development by various mechanisms. Both the HBc and HBx viral proteins are able to interfere with the host cell epigenetic machinery in different ways. While the role of HBc has not yet been defined in detail [37], HBx has been shown to have multiple effects on the host epigenetic machinery, including interaction with DNMTs, HMTs, CBP/P300 complex possessing HAT activity as well as ncRNAs, to modulate the expression of host genes associated with HCC. Epidrugs are in clinical use for hematological malignancies and are being evaluated for other diseases, including viral infections. Since the regulation of HBV cccDNA activity is governed by epigenetic mechanisms, epidrugs may hold potential for treatment of HBV infection (reviewed in [19,139,140]). Their use, alone or in combination with anti-HBV nucleos(t)ide analogs, in HBV patients as well as the rationale for their inclusion in new therapeutic combinations also including immune interventions is still unclear. No clinical data on epidrugs in HBV-related HCC are available at present. Given their potential positive or negative effects on HBV transcription, the effects of epidrugs in HBV-related HCC need to be carefully addressed.

## Figures and Tables

**Table 1:** Past and present clinical trials of epidrugs for Hepatocellular carcinoma (HCC).

Compound	Epidrug Target ^1^	Stage of Development	Clinical Trial
Belinostat	HDAC	Phase 1/2	NCT00321594
Decitabine + chemo-/immunotherapy	DNMT	Phase 1/2	NCT01799083
Guadecitabine + durvalumab	DNMT	Phase 1	NCT03257761
Guadecitabine + sorafenib + oxaliplatin	DNMT	Phase 2	NCT01752933
Resminostat + sorafenib	HDAC	Phase 1/2	NCT00943449

^1^ HDAC: histone deacetylase; DNMT: DNA methyltransferase.

**Table 2:** Epidrugs targeting host cell epigenetic alterations in HBV-associated HCC model systems.

Compound	Target ^1^	Model System	Reference
5-aza-2′-deoxycytidine (decitabine)	DNMTs	Cell lines	[130]
Nicotinamide	Sirt1	HBx transgenic mice	[66]
Resveratrol	Sirt1	Cell line, Huh7-HBx xenograft mice, HBx transgenic mice	[66,131,132,133]
Suberoylanilide hydroxamic acid (SAHA)	HDACs	Cell lines, pre-S 2 mutant LHBS transgenic mice	[129,134]
Trichostatin A (TSA)	HDACs	Cell lines	[135]
WDR5-0103	WDR5	Cell lines, MHCC97H and Huh7-HBx xenograft mice	[82]

^1^ DNMT: DNA methyltransferase; Sirt1: sirtuin 1; HDAC: histone deacetylase; WDR5: WD repeat domain 5.
